# A retrospective study of distribution of HIV associated malignancies among inpatients from 2007 to 2020 in China

**DOI:** 10.1038/s41598-021-03672-3

**Published:** 2021-12-21

**Authors:** Fang Wang, Pan Xiang, Hongxin Zhao, Guiju Gao, Di Yang, Jiang Xiao, Ning Han, Liang Wu, Hongyuan Liang, Liang Ni, Yujiao Duan, Qiuhua Xu, Meiling Chen, Fujie Zhang

**Affiliations:** 1 Clinical and Research Center of Infectious Diseases, Beijing Ditan Hospital, Capital Medical University, Beijing, China; 2 Department of Critical Care Medicine, Beijing Ditan Hospital, Capital Medical University, Beijing, China; 3 The Medical Statistic Department, Beijing Ditan Hospital, Capital Medical University, Beijing, China

**Keywords:** HIV infections, Cancer epidemiology

## Abstract

HIV-associated malignancies are responsible for morbidity and mortality increasingly in the era of potent antiretroviral therapy. This study aimed to investigate the distribution of HIV-associated malignancies among inpatients, the immunodeficiency and the effect of antiretroviral therapy (ART) on spectrum of HIV-associated malignancies. A total of 438 cases were enrolled from 2007 to 2020 in Beijing Ditan Hospital. Demographic, clinical and laboratory data, managements, and outcomes were collected and analyzed retrospectively. Of 438 cases, 433 were assigned to non-AIDS-defining cancers (NADCs) (n = 200, 45.7%) and AIDS-defining cancers (ADCs) (n = 233, 53.2%), 5 (1.1%) with lymphoma were not specified further. No significant change was observed in the proportion of NADCs and ADCs as time goes on. Of NADCs, lung cancer (n = 38, 19%) was the most common type, followed by thyroid cancer (n = 17, 8.5%). Patients with ADCs had lower CD4 counts(104.5/μL vs. 314/μL), less suppression of HIVRNA(OR 0.23, 95%CI 0.16–0.35) compared to those with NADCs. ART did not affect spectrum of NADCs, but affect that of ADCs (between patients with detectable and undetectable HIVRNA). ADCs remain frequent in China, and NADCs play an important role in morbidity and mortality of HIV positive population.

## Introduction

Human immunodeficiency virus (HIV) infection is known as one of risk factors to develop malignancies^1,2^. HIV-associated malignancies refer to a wide range of cancers with increased incidence in HIV-positive population, including AIDS-defining cancers (ADCs) and non-AIDS-defining cancers (NADCs). A surge of Kaposi’s sarcoma (KS) was documented in the early stage of AIDS epidemic with an unusually aggressive clinical course compared to KS among general population. Subsequently, non-Hodgkin lymphoma (NHL) and invasive cervical carcinoma (ICC) were added as ADCs^3^. Multiple factors contribute to development of malignancies including immunodeficiency, direct effect of HIV on various cellular process, coinfection with oncogenic organisms, risk behaviors (e.g., tobacco or alcohol use), environmental oncogenic factors and possibly antiretroviral therapy^4,5^.

The availability of protease inhibitors ushered in era of potent combined antiretroviral therapy (cART) since 1996. cART leads to immune restoration by suppression of HIV viral replication and normalization of CD4 lymphocyte which could decrease the incidence of opportunistic infections and risk of development of KS and some types of NHL. In the era of potent ART, the life expectancy of people living with HIV/AIDS (PLWHA) has been improved dramatically and the spectrum of malignancies among PLWHA has changed as well^6,7^. Of ADCs, KS and selected types of NHL develop most often among advanced immunodeficient patients, which incidence was 30% or more in patients of acquired immunodeficiency syndrome (AIDS) before introduction of ART^8^. As the incidence of those malignancies has decrease substantially with the wide use of cART^9^, a relative increase of NADCs were observed in HIV positive population compared with the general population, including Hodgkin lymphoma, melanoma, liver, lung, oropharyngeal, and colorectal cancers^10–13^. NADCs contribute to an increasing morbidity and mortality in HIV positive population^14,15^. PLWHA were often reported to be diagnosed with advanced-stage NADCs (e.g., melanoma and cancers of the oral cavity, liver, female breast, prostate, and thyroid) and to experience higher mortality than HIV negative population^16^. Malignancies among PLWHA often differ from those among general population, such as earlier age at onset, more aggressive clinical course, and/or atypical pathology (higher tumor grade), thus may lead to poorer outcome, higher rate of relapse, worse treatment response, and may require additional consideration of screening and treatment^17,18^. In high-income countries, malignancies have been reported to be one of the leading causes of death among HIV positive population^13,19^. However, there have been limited published studies about HIV-associated malignancies available in China.

In this study, we examined distribution of malignancies among HIV positive population in China, the demographic and clinical characteristics, the status of immunodeficiency among different types of malignancies, and effect of cART on spectrum of HIV-associated malignancies.

## Methods

### Study population

A total of 438 patients of HIV/AIDS with malignancies within 14 years from 2007 to 2020 were observed in Beijing Ditan Hospital, a tertiary care center in Beijing, China. These patients came from all over China’s mainland except Hainan, Tibet, Ningxia, Hongkong, and Macao. Patients with a foreign nationality, less than 18 years of age, or malignancies more than 2 years prior to HIV diagnoses were excluded. The patients were assigned to two groups of NADCs and ADCs based on association of malignancies and HIV infection.

### Diagnosis

HIV positive clients in Beijing Ditan Hospital have been screened and monitored properly for malignancies by symptoms, physical examination, imaging tests, or tumor markers. Patients at risk of specific cancers were paid more attention, such as the aged, or those coinfected with oncogenic organisms. A biopsy by fine needle aspiration biopsy (FNAB), CT or ultrasound-guided puncture, or surgical procedure etc. was recommended for histopathological diagnosis when there were suspected lesions.

Of 438 patients in this study, 427 had confirmed histopathological diagnoses by biopsy, although 11 liver cancers were confirmed by compatible clinical symptoms, typical imaging findings and elevated specific marker (i.e., Alpha-fetoprotein, AFP, especially AFP-L3)^20^.

HIV diagnoses were established by western blot test consistent with professional standards^21^.

### Data collection

The demographic data including gender, age (years), marriage, and routes of HIV transmission, clinical date including course of tumor (months), CD4 count (/μL), HIVRNA (copies/ml), whether on ART or under suppression of HIVRNA, managements and outcomes of tumor were collected.

### Definition

The term of ADCs typically refers to KS, NHL of high-grade pathologic type and of B cell or unknown immunologic phenotype, and ICC^22^. AIDS-defining NHLs include primary central nervous system lymphoma (PCNSL), Burkitt’s lymphoma (BL), diffuse large B cell lymphoma (DLBCL), plasmablastic lymphoma, and primary effusion lymphoma (PEL)^23^. Lung cancer, hepatic carcinoma, gastric cancer, anorectal cancer, leukemia, etc. are categorized as NADCs.

### Statistical analysis

Data were analyzed by SPSS 20 (SPSS Institute, Chicago IL, USA). Data followed a normal distribution (age and HIVRNA) were shown as arithmetic mean ± standard deviation, t test was conducted, while those followed a skew distribution were shown as median and interquartile range (IQR), Wilcoxon rank sum test was used to compare between two samples of NADCs and ADCs. Kruskal–Wallis test was used to compare CD4 count among different types of HIV-associated malignancies. Pearson χ^2^ test was performed to compare qualitative data. Log 10 of HIVRNA was analyzed, but it was shown as the original HIVRNA (copies/ml).

In this study, *p* value < 0.05 was considered to be statistically significant.

### Ethical consideration

This was a retrospectively designed study that performed in Beijing Ditan Hospital, Capital Medical University. The study protocol was approved by Ethics Committee of Beijing Ditan Hospital, Capital Medical University, which complied with principles of Declaration of Helsinki. All clinical and laboratory data of the participants were collected and analyzed anonymously and written informed consent was not required due to retrospective study using deidentified clinical information.

## Results

### Overview

Among 438 HIV/AIDS patients with malignancies, 433 patients had a confirmed diagnosis that could be assigned to either NADCs (n = 200, 45.7%) or ADCs (n = 233, 53.2%), while 5 (1.1%) patients with lymphoma were hard to be specified further.

**Figure 1:**
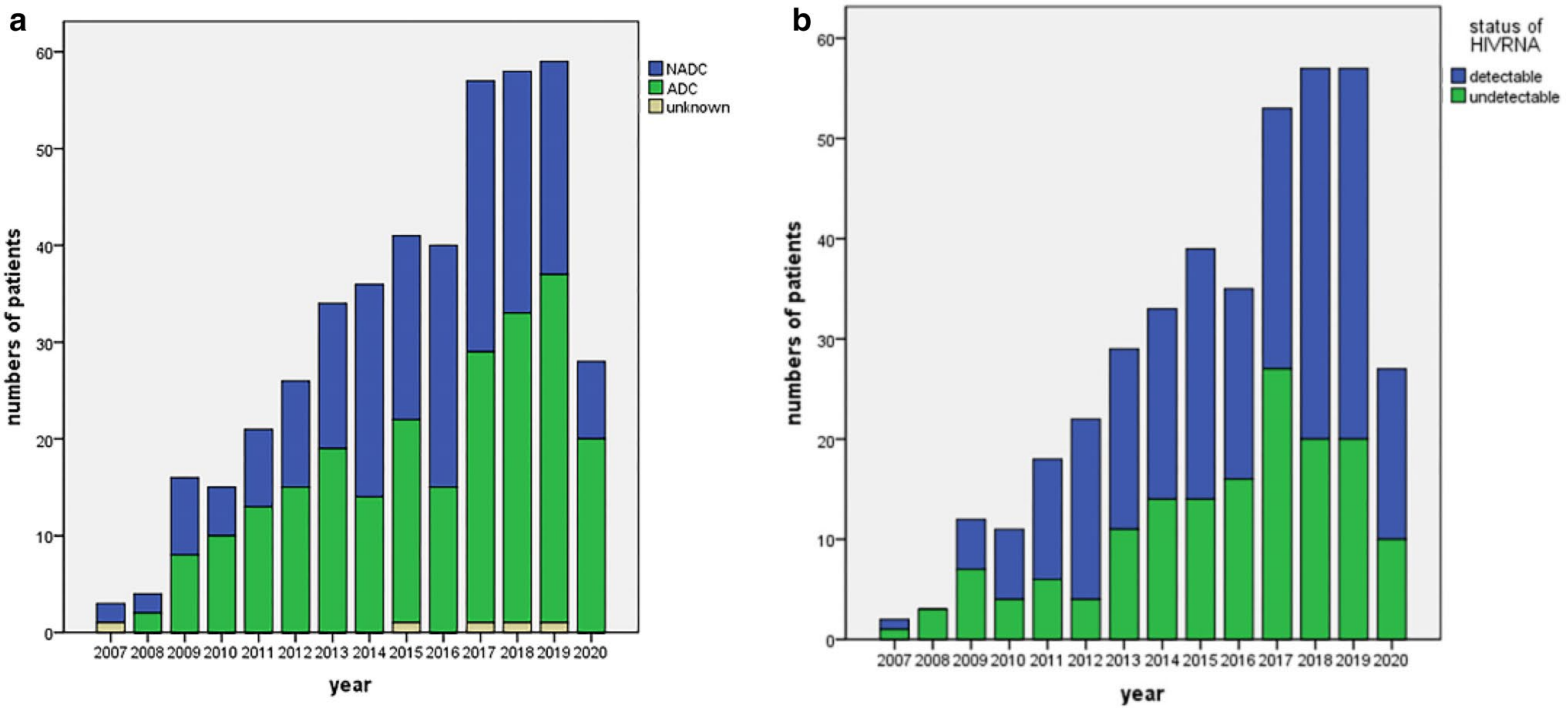
Trend in malignancies among HIV-infected patients and status of HIVRNA suppression (created by SPSS 20). (**a**) Trend in malignancies among HIV-infected patients in Beijing Ditan Hospital (according to calendar year, categorized as ADCs, NADCs, and uncertain ones). (**b**) Trend in status of HIVRNA suppression (detectable/undetectable) among HIV-infected patients according to calendar year.

Proportions of NADCs were 28.6% to 66.7%, while those of ADCs were 0% to 71.4% according to the calendar year (Fig. 1a). There was no statistical difference in trend of proportions between NADCs and ADCs as time goes on in our study (χ^2^ = 18.253, *p* = 0.148).

Patients who achieved HIV suppression (39.7%) were not in majority of this population, and the proportion of patients with an undetectable HIVRNA was not increasing during the observation period (Fig. 1b). No statistical difference was shown in proportion of HIVRNA status (detectable/undetectable) according to the calendar year (χ^2^ = 17.155, *p* = 0.192).

**Figure 2:**
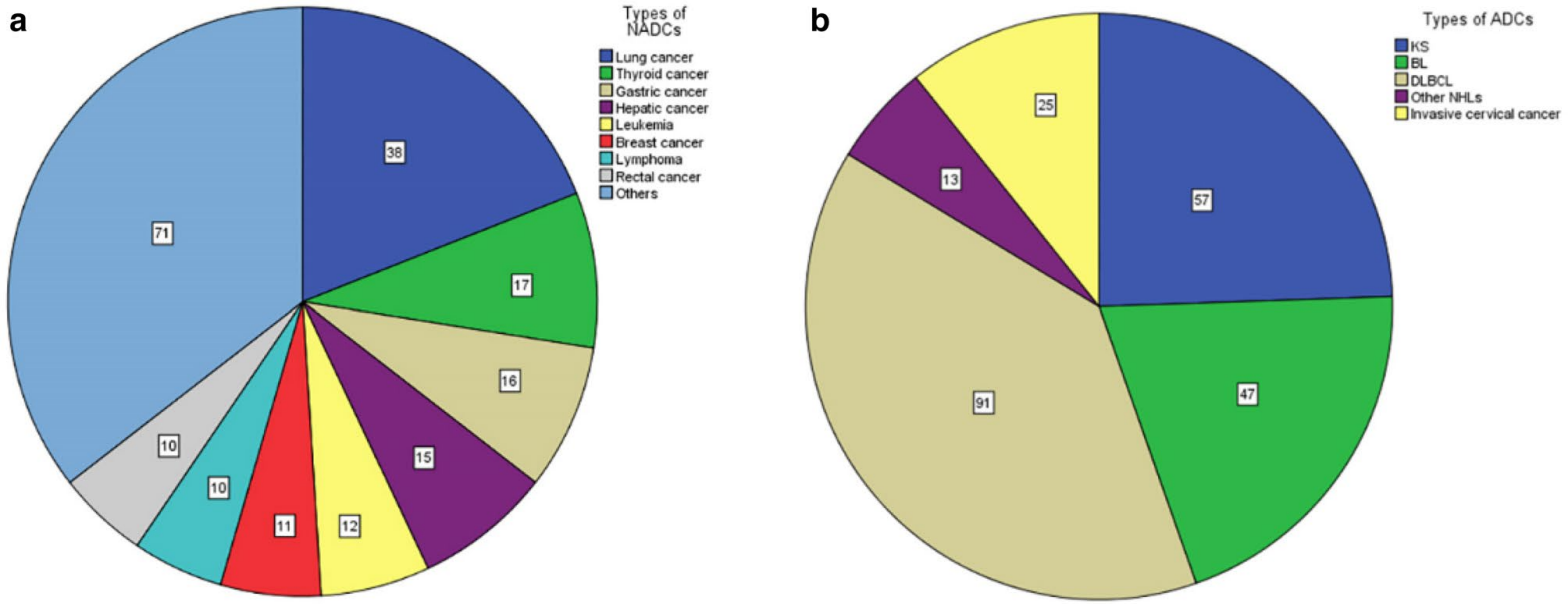
Types of malignancies diagnosed in Beijing Ditan Hospital, 2007–2020. (The numbers in the figure denoted those of each type of cancer). (**a**) Common types of NADCs. Uncommon NADCs (less than 5%) were summed as others. (**b**) Types of ADCs. *KS* Kaposi’s sarcoma, *BL* Burkitt’s lymphoma, *DLBCL* diffuse large B cell lymphoma.

Of 200 patients with NADCs, lung cancer (n = 38, 19%) was the most common, followed by thyroid cancer (n = 17, 8.5%), gastric cancer (n = 16, 8%), hepatic carcinoma (n = 15, 7.5%), leukemia (n = 12, 6%), breast cancer (n = 11, 5.5%), rectal cancer (n = 10, 5%), and lymphoma (n = 10, 5%). Uncommon NADCs including esophagus cancer (n = 8, 4%), Colon cancer (n = 8, 4%), brain tumor (n = 7, 3.5%), Hodgkin lymphoma (n = 5, 2.5%), Renal cancer (n = 5, 2.5%), cholangiocarcinoma (n = 4, 2%), Pancreatic cancer (n = 3, 1.5%), Anal cancer (n = 3, 1.5%), Ovarian cancer (n = 3, 1.5%), Bone tumor (n = 3, 1.5%), Laryngocarcinoma (n = 2, 1%), Oral carcinoma (n = 2, 1%), Prostate cancer (n = 2, 1%), Skin cancer (n = 2, 1%), Myeloid sarcoma (n = 1, 0.5%), Nasopharynx cancer (n = 1, 0.5%), Gallbladder carcinoma (n = 1, 0.5%), Peritoneal carcinoma (n = 1, 0.5%), Ampulla cancer (n = 1, 0.5%), Endometrial cancer (n = 1, 0.5%), Penis carcinoma (n = 1, 0.5%), Bladder cancer (n = 1, 0.5%), Testiculoma (n = 1, 0.5%), Leiomyosarcoma (n = 1, 0.5%), metastatic cancers of unidentified origin (n = 4, 2%) were observed (Fig. 2a).

Of 233 ADCs, 151 cases of NHLs (64.8%), 57 cases of KS (24.5%), and 25 cases of invasive cervical cancer (10.7%) were described in this study. Of NHLs, there were 47 cases of BL (20.2%), 91 cases of DLBCL (39.1%), and 13 cases of other NHLs (5.6%) (Fig. 2b).

Compared two groups of NADCs and ADCs, patients with ADCs were at younger ages when malignancies onsets (39.83 ± 11.56 years vs. 48.33 ± 13.1 years), had shorter term from identification of HIV infection to diagnoses of malignancies (4 months vs. 36 months), lower CD4 counts (104.5/μL vs. 314/μL), and less suppression of HIVRNA (OR 0.23, 95%CI 0.16–0.35). The outcomes between two groups of NADCs and ADCs had no statistical difference (Table 1).

**Table 1:** Characteristics of patients with malignancies and HIV infection.

	Total (n = 438)	NADCs (n = 200)	ADCs (n = 233)	Value	*p*
Gender(M/F)	355/83	157/43	193/40	χ^2^ 1.488	0.222
Age(y)	43.66 ± 12.99	48.33 ± 13.10	39.83 ± 11.56	Z − 6.834	0.000
Course of HIV (month)	12 (1–48) (n = 354)	36 (9.25–69) (n = 164)	4 (1–24) (n = 185)	Z − 6.135	0.000
Course of tumor(month)	2(1–6)	2(1–6)	2(1–4)	Z − 0.997	0.319
**Routes of transmission**					
MSM	72	16	55	χ^2^ 51.823	0.000
Heterosexual	22	6	16		
Sex (unknown)	22	2	18		
Blood transfusion	44	32	12		
Mother to child	1	0	1		
Unwilling to tell	276	143	131		
IDU	1	1	0		
CD4(/μL)	196.5 (69.5–375) (n = 420)	314 (164–495) (n = 191)	104.5 (29.25–256) (n = 224)	Z − 8.961	0.000
HIVRNA (copies/ml)	15,219.98 ± 26.95 (n = 330)	10,680.71 ± 20.42 (n = 146)	16,672.47 ± 26.92 (n = 180)	Z − 1.063	0.288
status of HIVRNA (undetectable/detectable) status of HIVRNA (undetectable/detectable)	157/241	103/73 (n = 176)	53/164 (n = 217)	χ^2^ 47.203	0.000
**Management**					
Surgery	75	56	19		
Chemotherapy	157	40	116		
Palliative	187	89	94		
Surgery + chemotherapy	19	15	4		
**Outcomes**					
Improved	190	89	100	χ^2^ 1606	0.205
Unimproved	178	89	86		
Death	70	22	47		

Data (age and HIVRNA) followed a normal distribution shown as arithmetic mean ± standard deviation, others followed a skew distribution shown as median and interquartile range(IQR).

### Immunodeficiency among different types of HIV-associated malignancies

**Figure 3:**
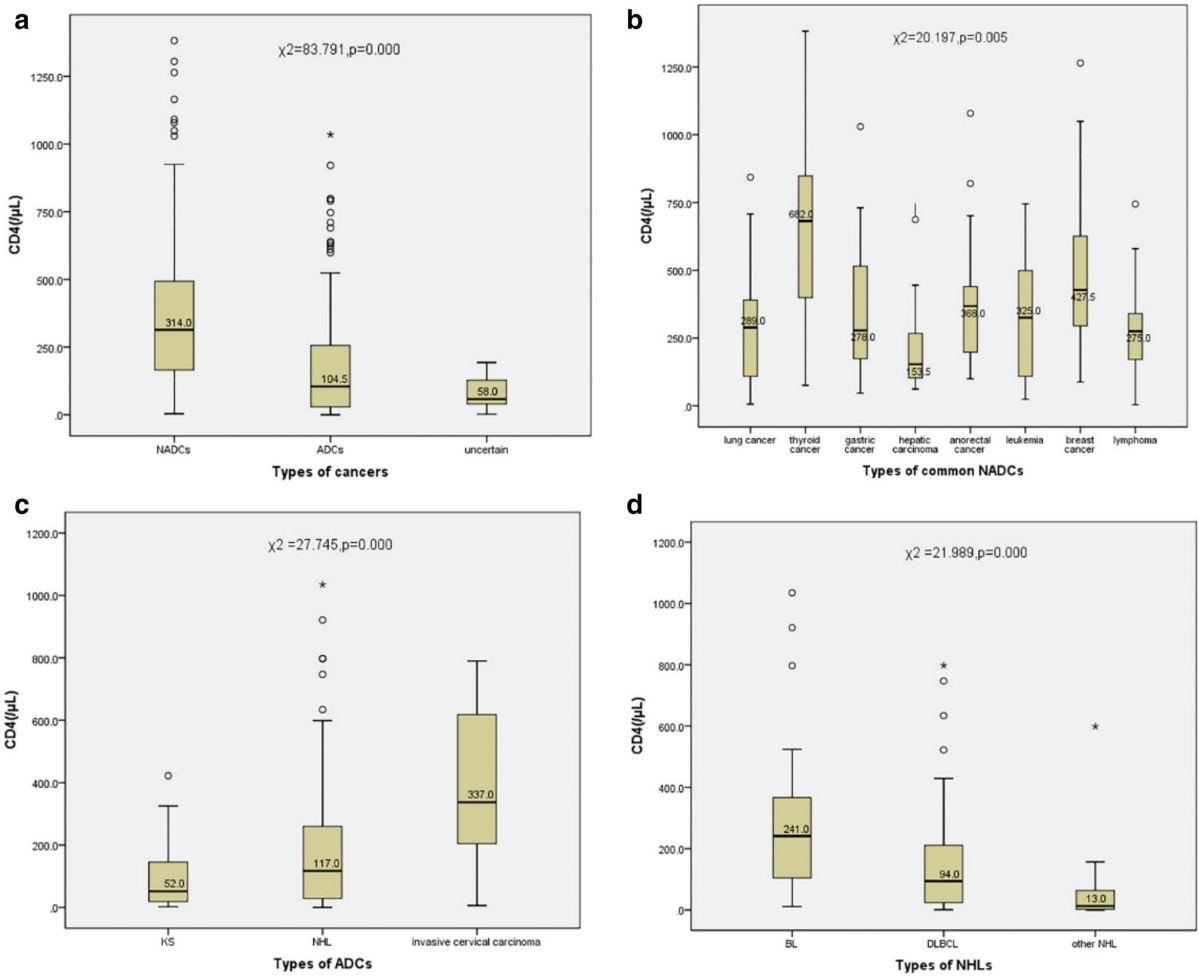
CD4 counts (median) in different types of HIV-associated malignancies.

The immune status of the patients with HIV-associated malignancies at the time of diagnosis were observed. The immunodeficiency differs among different types of HIV-associated malignancies (Fig. 3). The median CD4 counts was much higher in NADCs compared with those in ADCs (314/μL and 104.5/μL, respectively) (Fig. 3a). Patients with common NADCs were at relatively high CD4 level (Fig. 3b), while hepatic carcinoma was an exception (the CD4 median was 153.5/μL). Of ADCs, KS was most closely associated with immunodeficiency, which median CD4 count was 52/μL (Fig. 3c). The development of NHLs was also related to immunodeficiency, but second to KS, which median CD4 count was 117/μL (BL 241/μL, DLBCL 94/μL, other NHLs 13/μL, respectively) (Fig. 3d). Patients with ICC were less immunodeficient than those with KS or NHL, which median CD4 count was 337/μL (Fig. 3c).

### Effect of ART on spectrum and characteristics of HIV-associated malignancies

**Figure 4:**
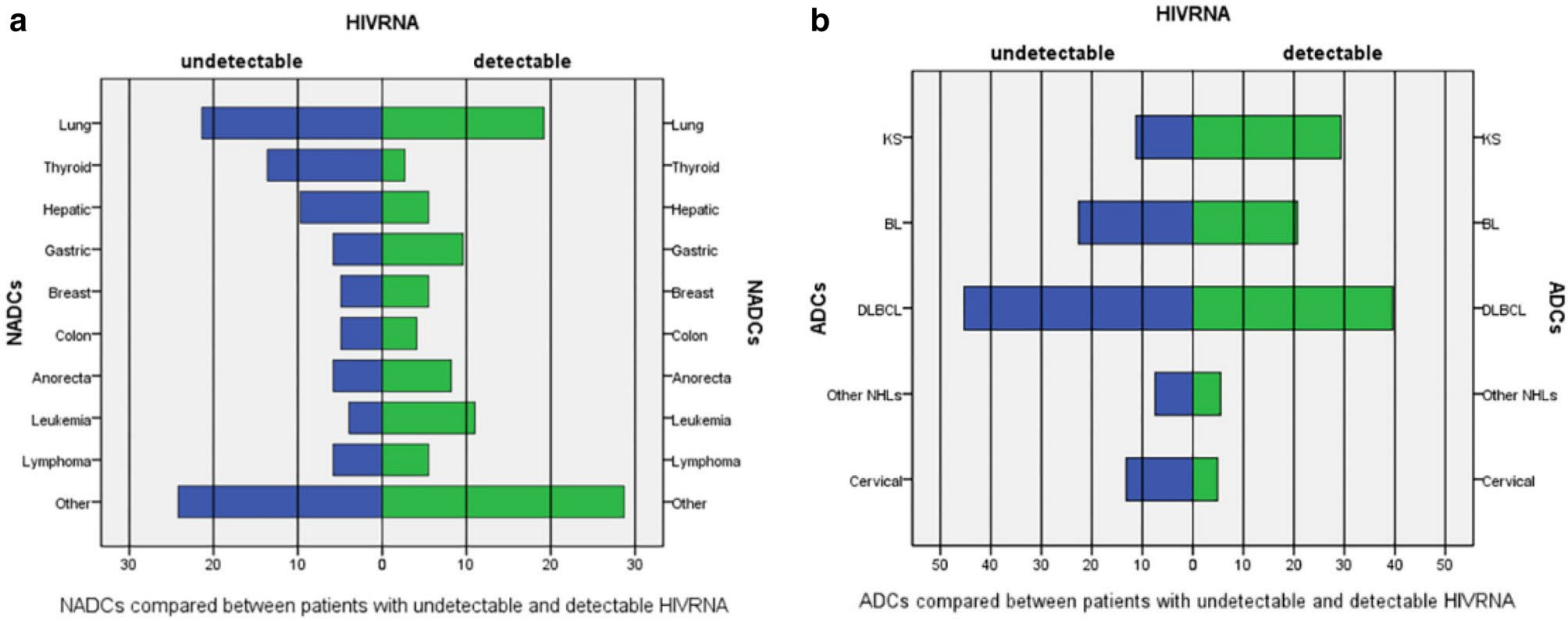
Spectrum of NADCs and ADCs stratified by status of HIVRNA.

To describe the effect of ART on HIV-associated malignancies, the participants were divided into two groups according to their HIVRNA, i.e., detectable and undetectable groups. The most common NADCs was lung cancer irrespective of whether HIVRNA is fully suppressed or not, and the proportions of different types of common NADCs between patients with detectable HIV viral load (VL) and undetectable HIV VL were not different statistically (χ^2^ = 9.427, *p* = 0.223) (Fig. 4a).

ART affected spectrum of ADCs (Fig. 4b). The proportions of different types of ADCs between two groups were different statistically (χ^2^ = 9.869, *p* = 0.043). The proportion of KS was lower among patients with an undetectable HIV VL (χ^2^ = 8.056, *p* = 0.005). Nevertheless, the proportions of different types of NHLs (BS, DLBCL, and other NHLs) showed no statistical difference between two groups (χ^2^ = 0.113, *p* = 0.945).

Patients with a detectable HIV VL had shorter course of HIV compared with those with an undetectable VL (both NADCs and ADCs), shorter course of tumor among ADCs (2 month vs. 3 month). The outcomes had no statistical difference irrespective of HIVRNA status in either NADCs or ADCs group (Table 2).

**Table 2:** Characteristics of NADCs and ADCs stratified by status of HIVRNA.

	NADCs				ADCs			
	Status of HIVRNA		Value	*p*	Status of HIVRNA		Value	*p*
	Detectable (n = 73)	Undetectable (n = 103)			Detectable (n = 164)	Undetectable (n = 53)		
Gender(M/F)	64/9	78/25	χ^2^ 3.910	0.048	142/22	45/8	χ^2^ 0.095	0.758
Age(y)	52 ± 12.62	47.19 ± 13.04	t 1.557	0.121	38.18 ± 10.85	41.55 ± 11.49	t − 1.935	0.054
Course of HIV(month)	6(0.5–3.6) (n = 53)	48(18–84) (n = 95)	Z − 5.449	0.000	2(0.6–12) (n = 129)	24(4.5–60) (n = 45)	Z − 5.312	0.000
Course of tumor(month)	2(1–6) (n = 73)	2(1–6) (n = 103)	Z − 0.315	0.753	2(1–4) (n = 164)	3(1.5–6.5) (n = 53)	Z − 2.172	0.030
CD4(/μL)	287.5(127–429.35) (n = 72)	352(170.5–687.5) (n = 103)	Z − 2.512	0.012	81(19.25–200.75) (n = 164)	203.5(92.5–340) (n = 48)	Z − 4.671	0.000
Improved	29	49	χ^2^ 1.508	0.219	70	23	χ^2^ 0.161	0.689
Unimproved	33	44			58	21		
Died	11	10			36	9		

## Discussion

HIV positive population are at risk to develop malignancies, including NADCs or ADCs. More cases of ADCs were documented than NADCs from 2007 to 2020, demonstrated ADCs remained frequent in China. In this retrospective study, no significant change was seen in the proportion of ADCs and NADCs according to the calendar year (Fig. 1a). The status of HIV suppression was supposed to be associated with this trend in our institution (Fig. 1b). China National Free Antiretroviral Treatment Programme (NFATP) which was scaled up in 2003 has benefited PLWHA widely^24^, and the study population was within the era of cART. However, there are patients that remain unaware of their HIV status currently. Patients who presented with ADCs as their initial symptoms of HIV/AIDS were often at advanced stage of HIV infection, characterized as low CD4 levels, suggesting long-term underlying HIV infection rather than recent infection. Of those cases, short term (mean of 4 months) from HIV identification to ADCs diagnosis was observed, mainly because many of the patients did not realize they were infected with HIV until malignancies onset. Therefore, the popularization of correct understanding of HIV/AIDS, especially to the population at high risk is important for early diagnosis of HIV infection and access to appropriate care, including ART.

The CD4 counts of patients with NADCs were at higher levels than those of patients with ADCs and ART did not affect spectrum of NADCs significantly. Meanwhile, the relatively long course (mean of 36 months) from HIV confirmation to malignancies diagnosis and higher proportion of patients with fully suppressed HIVRNA (Table 1) could be attributed to the effect of cART. NADCs are responsible for an increasing morbidity and mortality in the modern ART era, probably because the extension of lifespan made PLWHA at higher risk for developing a variety of malignancies. Yanik et al. reported that incidence of NADCs were not observed associated with the time of ART use^25^. Therefore, prevention of oncogenic factors, e.g., vaccination for oncogenic virus (e.g., HBV, HCV, HPV) or behaviors modification, e.g., smoking cessation should be introduced.

Hodgkin’s lymphoma, gastrointestinal cancer, hepatic carcinoma, lung cancer, HPV-related cancers were reported commonly among HIV positive population^8,26,27^. There was some difference in prevalence of NADCs in this study. Lung cancer was the most frequent NADCs in our subject, followed by thyroid cancer. Data about prevalence of thyroid cancer were controversial^28,29^. Screening of thyroid cancer is easy to implement by ultrasound in order to provide a rapid treatment.

KS (n = 57, 13.0%) and NHL (n = 151, 34.5%) were in the majority of ADCs. Of ADCs, KS developed with the lowest CD4 counts, NHLs come second. Both of them are related to advanced immunodeficiency. Patients with ICC often had higher CD4 counts than those with KS or NHLs, similar to NADCs. The prevalence of KS was inversely correlated with CD4 count as well known, and had a decrease dramatically as a consequence of fully suppressed HIVRNA by the widespread use of cART. By contrast, prevalence of cervical cancer had not declined significantly irrespective of cART^9,30^. HPV vaccine helps to prevent development of cervical cancer, however.

Patients with ADCs were younger at onset of malignancies and had more rapid progression from HIV infection to malignancies diagnosis illustrated more aggressive clinical course compared with NADCs; lower CD4 counts and less suppression of HIVRNA that representative of advanced immunodeficiency.

Previous studies described that HIV-positive patients with malignancies had worse outcomes than those of HIV-negative^31,32^. However, recent researches have illustrated that HIV infection does not affect prognoses of selected malignancies in the potent era of ART^33–35^. The outcomes had no statistical difference between two groups of NADCs and ADCs and irrespective of HIVRNA status in either NADCs or ADCs group. Moreover, 89 cases of NADCs and 100 cases of ADCs improved through aggressive treatments revealed that appropriate approaches to malignancies managements improved the outcome substantially. With the incremental refinements in malignancies managements, the overall survival (OS) of many types of cancer were improved significantly^36–40^. Clinicians should promote quality cancer treatment as well as cART. Consultation with surgeons or oncologists for surgery or chemoradiotherapy is imperative. Of note, drug-drug interactions between many chemotherapeutic and antiretroviral agents need to be taken into consideration given the possibility of either increased drug toxicity or decreased efficacy.

We also advocate that cancer screening and prevention be counselled to each patient with HIV infection, and be conducted throughout their life. Moreover, treat strategies should be improved since better prognosis of considerable types of malignancies is conceivable.

There were limitations in our analysis. This was a single center study, and most patients came from northern China, so the spectrum and trend of malignancies could not represent those in the whole country. Biases existed in this observational study, because the study population were not a random one. Patients were admitted in different departments (infectious, oncology, surgery, etc.) where clinicians laid emphasis on different aspects, so that some clinical data were not sufficient.

## Conclusion

Malignancies is a major issue that responsible for morbidity and mortality among PLWHA, although in the modern era of ART. Both NADCs and ADCs are frequent in China currently. Consequently, earlier diagnosis and quality care of malignancies are essential as well as cART for better prognosis.

## Abbreviations

HPV: Human papillomavirus
HBV: Hepatitis B virus
HCV: Hepatitis C virus
MSM: Men who have sex with men
IDU: Intravenous drug user
